# Valorization of Hemp-Based Packaging Waste with One-Pot Ionic Liquid Technology

**DOI:** 10.3390/molecules28031427

**Published:** 2023-02-02

**Authors:** Julius Choi, Alberto Rodriguez, Blake A. Simmons, John M. Gladden

**Affiliations:** 1 Deconstruction Division, Joint BioEnergy Institute, 5885 Hollis Street, Emeryville, CA 94608, USA; 2 Department of Biomaterials and Biomanufacturing, Sandia National Laboratories, 7011 East Avenue, Livermore, CA 94550, USA; 3 Biological Systems and Engineering Division, Lawrence Berkeley National Laboratory, 1 Cyclotron Road, Berkeley, CA 94720, USA

**Keywords:** biomass pretreatment, cholinium lysinate, *Rhodosporidium toruloides*, bisabolene

## Abstract

The range of applications for industrial hemp has consistently increased in various sectors over the years. For example, hemp hurd can be used as a resource to produce biodegradable packaging materials when incorporated into a fungal mycelium composite, a process that has been commercialized. Although these packaging materials can be composted after usage, they may present an opportunity for valorization in a biorefinery setting. Here, we demonstrate the potential of using this type of discarded packaging composite as a feedstock for biofuel production. A one-pot ionic liquid-based biomass deconstruction and conversion process was implemented, and the results from the packaging material were compared with those obtained from untreated hemp hurd. At a 120 °C reaction temperature, 7.5% ionic liquid loading, and 2 h reaction time, the packaging materials showed a higher lignocellulosic sugar yield and sugar concentrations than hemp hurd. Hydrolysates prepared from packaging materials also promoted production of higher titers (1400 mg/L) of the jet-fuel precursor bisabolene when used to cultivate an engineered strain of the yeast *Rhodosporidium toruloides*. Box–Behnken experiments revealed that pretreatment parameters affected the hemp hurd and packaging materials differently, evidencing different degrees of recalcitrance. This study demonstrated that a hemp hurd-based packaging material can be valorized a second time once it reaches the end of its primary use by supplying it as a feedstock to produce biofuels.

## 1. Introduction

Lignocellulosic biomass can be a renewable carbon resource for the production of fuels and chemicals to replace non-renewable fossil carbon sources such as natural gas, oil, and coal [1]. Lignocellulosic biomass can be converted into a wide range of chemicals and energy carriers through a closed-loop biorefinery [2,3]. Despite decades of scientific and technological advances in this field, many technical challenges associated with the recalcitrance of lignocellulose to deconstruction into fermentable carbon still hamper the establishment of economically feasible biorefineries. These include the lack of efficient and inexpensive processes to depolymerize all components of biomass and convert them to fuels and chemicals at high yields by microbial fermentation [4,5]. To improve the accessibility of the biomass carbohydrate polymers to hydrolytic enzymes, a pretreatment step is frequently required. Numerous pretreatment technologies have been developed, including physical methods (e.g., milling, grinding, irradiation, and sonication) [6,7], chemical methods (e.g., alkali, acid, oxidizing agents, organic solvents, ionic liquids, and deep-eutectic solvents) [8,9], combined physico-chemical methods (e.g., hydrothermolysis) [10,11], or biological methods (e.g., bacteria and fungi) [12].

The discovery and implementation of biocompatible ionic liquids such as cholinium lysinate ([Ch][Lys]) provide a compelling alternative to the aforementioned approaches because of their high pretreatment efficiency and lower inhibitory effect to enzymes and microbes [13,14,15]. These features have allowed the development of a one-pot reaction system where all the process steps, such as pretreatment, enzymatic hydrolysis and fermentation can be consolidated into a single vessel without any separation, potentially reducing operating costs. Our group has demonstrated the applicability of this process by generating hydrolysates with high concentrations of monomeric sugars and organic acids from several feedstocks like grasses, hardwoods, and softwoods, and converting them to terpene-based jet-fuel molecules using engineered strains of the yeast *Rhodosporidium toruloides* [16,17,18]. Nevertheless, it is important to expand the range of lignocellulosic feedstocks used in this process to evaluate its versatility to advance towards the goal of developing a truly lignocellulosic feedstock-agnostic biorefinery.

Hemp is an attractive crop due to its fast growth, bioremediation potential, and diverse agricultural applications, including the production of natural fibers, grains, essential oils, and other commodities [19]. This biomass is composed of an outer fiber that represents approximately 30% of the weight and an inner core known as hurd that accounts for the remaining 70% [20]. The hemp fiber is utilized in the textile industry as insulation material and for the production of bioplastics in the automotive industry, while hemp hurd is used for low value applications such as animal bedding, concrete additives, or disposed of by combustion and landfill accumulation [21,22,23]. This indicates that approximately 70 wt% of hemp biomass has the potential to be valorized into higher-value products and applications, which would improve the economics of the hemp industry and increase its sustainability footprint to promote a green economy. Mycelium-based composites are emerging as cheap and environmentally sustainable materials generated by fungal growth on a scaffold made of agricultural waste materials [24]. The mycelium composite can replace foams, timber, and plastics for applications like insulation, packaging, flooring, and other furnishings [24]. For example, the company Ecovative Design LLC (Green Island, NY, USA) produces a foam-like packaging material made of hemp hurd and fungal mycelia, which is fully compostable. Anticipating the possibility of an increased demand of eco-friendly packaging materials in the near future, we are interested in evaluating the feasibility of diverting this used packing material away from landfills or composting facilities towards higher value applications, such as feedstock for biofuels. It is known that fungal enzymes can reduce the recalcitrance of the biomass to deconstruction [25,26], likely through modification of polysaccharides and lignin in plant biomass. Therefore, we hypothesized that the mycelium composite material could be more easily deconstructed and converted into higher value fuels and chemicals than the raw hemp hurd.

In this study, hemp hurd and the mycelium-based packaging material were tested as biomass feedstocks for the production of the jet-fuel precursor bisabolene, using a one-pot ionic liquid technology and microbial conversion. First, we examined the deconstruction efficiency of the packaging material compared to hemp hurd, when subjugated to a one-pot ionic liquid pretreatment process. Second, the influence of the pretreatment process parameters on the sugar yields was investigated by using a Box–Behnken statistical design. Finally, the generated hydrolysates were fermented to evaluate the bioconversion of the depolymerized components by a bisabolene-producing *R. toruloides* strain.

## 2. Results and Discussion

### 2.1. Biomass Composition

The composition of the hemp hurd and packaging material was determined as shown in Table 1. The total extractives of the hemp hurd and packaging material comprised 8.3 and 14.7% of the biomass, respectively. The higher extractive content of the packaging material may be a result of the fungal (mycelium) growth stage in the packaging construction process. For the polysaccharide content, hemp hurd had higher glucan (30.3%) and xylan (13.5%) contents than the glucan (28.6%) and xylan (11.9%) content of the packaging material. Combining glucan and xylan content, the total fermentable sugars of the hemp hurd and packaging material was 43.7% and 40.4% of the hemp hurd biomass, respectively. This indicates that a small fraction of the polysaccharides may have been consumed and converted into extractives during mycelial growth. However, both types of biomass contain a substantial amount of polymeric carbohydrates that can be depolymerized into simple sugars for fermentation. The lignin content for both materials was the same (22.4%); however, it is possible that the mycelial growth in the packaging material could have altered the structure of lignin and made the polysaccharides more accessible to hydrolysis [27,28,29]. We used the one-pot ionic liquid process on hemp hurd and package materials to test this hypothesis.

### 2.2. Hydrolysate Generation Using a One-Pot Ionic Liquid Process

**Figure 1:**
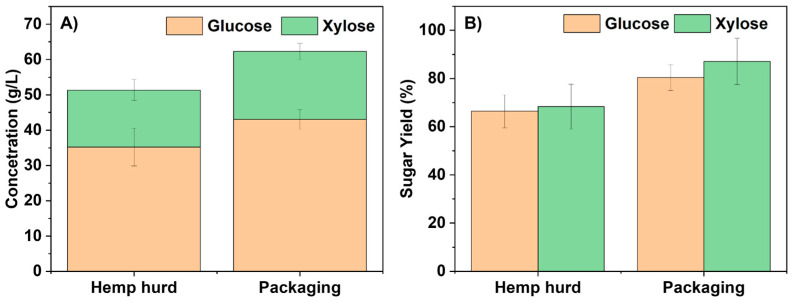
(**A**) glucose and xylose concentration and (**B**) glucose and xylose yield from enzymatic hydrolysis of [Ch][Lys]-pretreated hemp hurd and packaging materials.

The raw hemp hurd and packaging material were deconstructed into fermentable sugars under the same reaction conditions using 10 wt% [Ch][Lys] and 20 wt% biomass loading. Fermentable sugar concentrations and yields are shown in Figure 1. We observed glucose and xylose concentrations from packaging materials of 43.0 ± 2.9 g/L and 19.3 ± 2.3 g/L, respectively, while hemp hurd released 35.2 ± 5.3 g/L and 16.2 ± 2.9 g/L. These represent glucose and xylose yields of 80.4 ± 5.4 and 87.1 ± 9.6% for the packaging material and 66.4 ± 6.8 and 68.3 ± 9.3% for hemp hurd, as shown in Figure 1B. These results indicate that the process used to generate the packaging material renders both cellulose and hemicellulose 10–20% more digestible than in raw hemp hurd, probably due to the decreased biomass recalcitrance caused by mycelium growth.

### 2.3. Biocompatibility of Hydrolysates

One of the bottlenecks for the efficient conversion of lignocellulosic hydrolysates is the presence of compounds generated during the pretreatment and enzymatic hydrolysis stages that are toxic to biofuel-producing microbes [30,31]. The degree of toxicity mainly depends on the type of biomass, pretreatment conditions, and the identity of the microorganism that will be used for fermenting the depolymerized substrates. Therefore, we performed a biocompatibility test with the hydrolysates prepared from hemp hurd and packaging materials, using an engineered strain of the yeast *R. toruloides* known to be tolerant to ILs and biomass-derived compounds, and convert glucose and xylose to the jet fuel precursor bisabolene [32,33].

**Figure 2:**
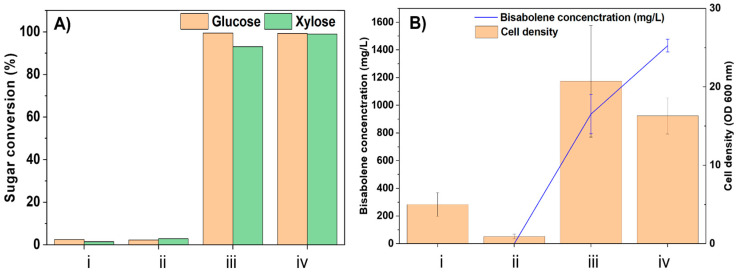
(**A**) sugar conversion and (**B**) cell density and bisabolene titer after fermentation of as-prepared hydrolysates from (i) hemp hurd and (ii) packaging material, and 50% diluted hydrolysates from (iii) hemp hurd and (iv) packaging material.

When the strain was inoculated directly in concentrated hydrolysates, negligible sugar consumption and very little growth was observed, as shown in Figure 2. Therefore, we prepared 50% diluted hydrolysates for further testing. Under these conditions, more than 90% of glucose and xylose conversion was observed in both hydrolysates, and the cells were able to grow and produce bisabolene (Figure 2). This result suggests that there is some degree of toxicity present in these hydrolysates. The utilization of hydrolysate with higher concentrations is beneficial for the economically feasible biorefinery development [34,35]. Therefore, other strategies such as hydrolysate culture adaptation or detoxification may be required to improve biocompatibility [36].

As shown in Figure 2B, the cell growth in 50% diluted hemp hurd hydrolysates (OD600 = 20) was higher than the growth observed in 50% diluted hydrolysates from packaging material (OD600 = 16). Nevertheless, the bisabolene titer produced in 50% diluted hydrolysates from the packaging was higher than 50% diluted hydrolysates from hemp hurd hydrolysates (1400 mg/L in 50% diluted hydrolysate from packaging vs. 600 mg/L in 50% diluted hydrolysate from hemp hurd). These results show that the hydrolysate with higher initial sugars resulted in higher bisabolene concentration, as expected, and the lower cell biomass in hydrolysates from the packaging material may be caused by growth inhibitors produced by mycelial growth during the packaging process.

### 2.4. Effect of Process Parameters on Sugar Yield and Optimization of Pretreatment Conditions

To optimize the pretreatment condition and investigate the effect of the process factors such as reaction time, ionic liquid loading, and reaction time, we employed a response surface methodology (RSM) (Table 2).

Tables S1 and S2 show the glucose and xylose yield from hemp hurd and packaging materials obtained from Box–Behnken-designed experiments. The glucose yield from hemp hurd ranged from 28.2% to 81.6%, and the packaging material ranged from 51.2% to 75%. Xylose yields from hemp hurd and packaging materials varied in the range of 22.7–90.5% and 51.8–80%, respectively. The quadratic regression models for glucose yield and xylose yields from hemp hurd and packaging materials are shown as Equations (1)–(4) below:

(1) $$Y_{glucose}^{Hemp\;hurd}=0.68+0.14X_{1}+0.053X_{2}-0.073X_{3}-0.125X_{1}X_{2}+0.05X_{1}X_{3}\;-0.01X_{2}X_{3}+0.11X_{1}^{2}-0.14X_{2}^{2}-0.18X_{3}^{2}$$

(2) $$Y_{xylose}^{Hemp\;hurd}=0.69+0.17X_{1}+0.036X_{2}-0.055X_{3}-0.099X_{1}X_{2}+0.043X_{1}X_{3}\;0.025X_{2}X_{3}+0.14X_{1}^{2}-0.14X_{2}^{2}-0.20X_{3}^{2}$$

(3) $$Y_{glucose}^{packaging}=0.74+0.051X_{1}+0.024X_{2}-0.01X_{3}-0.038X_{1}X_{2}+0.015X_{1}X_{3}\;-0.01X_{2}X_{3}-0.072X_{1}^{2}-0.052X_{2}^{2}-0.04X_{3}^{2}$$

(4) $$Y_{xylose}^{packaging}=0.80+0.05X_{1}+0.028X_{2}+0.027X_{3}-0.035X_{1}X_{2}-0.021X_{1}X_{3}\;-0.03X_{3}-0.055X_{1}^{2}-0.086X_{2}^{2}-0.055X_{3}^{2}\;$$

**Figure 3:**
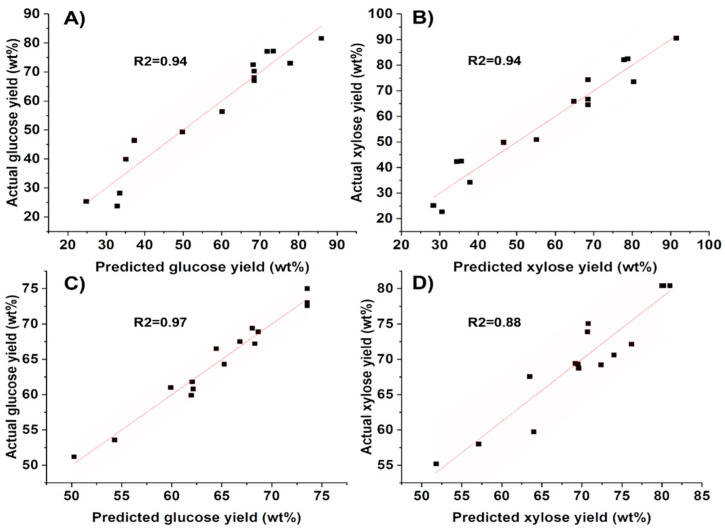
Actual yield versus predicted yield of glucose and xylose from (**A**,**B**) hemp hurd and (**C**,**D**) packaging material.

As shown in Figure 3 and summary of fit in Tables S3–S6, the models fitted well with experimental data with R2.

The optimum levels of parameters for glucose and xylose yields from packaging materials recommended by the model were: reaction temperature of 126 and 128 °C, reaction time of 2.1 and 2.0 h, and ionic liquid loading of 7.3% and 7.9%, corresponding to a predicted glucose and xylose yield of 74.6% and 81.7%. However, this optimal condition did not significantly improve the yields compared to the center point, even though the reaction conditions required a 4% higher temperature than the center point, a rather small difference in temperature. This result suggests that other process parameters such as agitation and biomass solid loading percentage should be tested for further improvement in the yield.

The model for hemp hurd found a saddle point instead of optimum levels, which means that the optimum process condition was not aligned within the current experimental conditions [37]. Further investigation into the different range of reaction conditions such as higher reaction temperature is required to optimize the reaction condition for hemp hurd. If operating with a limited budget and time, the reaction condition having the highest glucose and xylose yield can be chosen [37]. The highest glucose yield (81%) in the current reaction condition was obtained from hemp hurd at 140 °C, 1 h reaction time and 7.5% ionic liquid loading, which has higher severity in reaction condition than the optimized reaction condition of packaging materials. This result indicates that the reaction parameter affects the sugar yield differently according to the biomass type, implying that the biomass properties change by mycelium growth.

**Figure 4:**
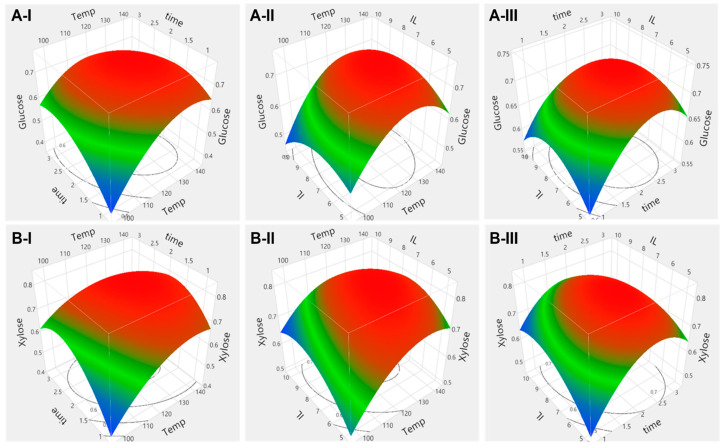
Surface response plot for the combined effect of (I) reaction time and temperature, (II) ionic liquid loading and temperature and (III) ionic liquid loading and reaction time on (**A**) glucose and (**B**) xylose yield from packaging material.

Regarding the packaging materials, the combined effects of reaction temperature, reaction time and ionic liquid loading on glucose yields are illustrated in Figure 4(A-I–A-III) and xylose yields in Figure 4(B-I–B-III). Response surface plots show that the glucose yield increased with the reaction temperature up to 133 °C with subsequent decrease in yield at a higher temperature. The xylose yields showed a similar trend. Additionally, the glucose and xylose yield increased with the reaction time up to 2 h and 7.5% ionic liquid loading. After those points, the glucose and xylose yield decreased, probably due to the loss of enzyme activity caused by the higher ionic liquid concentration [38]. Additionally, the longer reaction time and the higher ionic liquid concentration might facilitate the production of other compounds such as furan derivatives or organic acids, which inhibits the enzyme activity during the pretreatment [2,39]. Moreover, the production of other components probably led to a decrease in accessible carbohydrates to the enzyme [40]. Further tests may be necessary to improve the sugar yield. ANOVA results shown in Table S5 indicate that reaction temperature and reaction time has statistically significant effects on glucose yield (*p* < 0.0008 and 0.025, respectively), while ionic liquid loading was not significant (*p* > 0.2134). Additionally, the statistically significant interaction effects of reaction temperature with reaction time and ionic liquid loadings (*p* < 0.0012 and *p* < 0.0153) were confirmed. ANOVA results associated with xylose yield (Table S6) show that reaction temperature had a significant effect on the yield (*p* < 0.0318), while reaction time and ionic liquids had no effect (*p* > 0.1702 and 0.1768). Additionally, the interaction effect of reaction temperature with reaction time and ionic liquid loading was not significant, while the interaction effects of reaction time with ionic liquid were significant (*p* < 0.0185).

**Figure 5:**
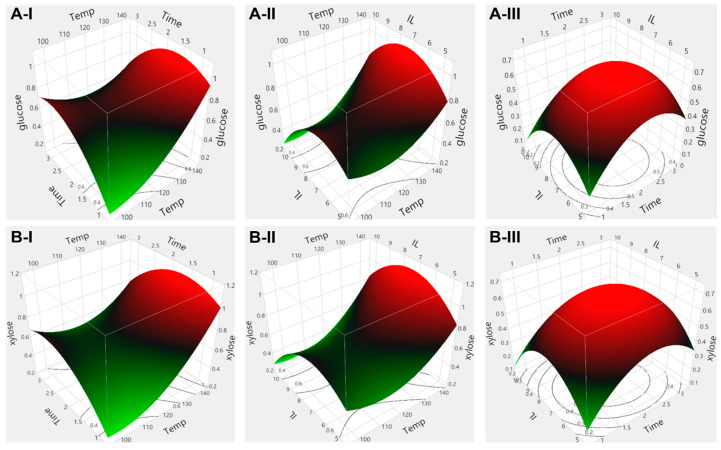
Surface response plot for the combined effect of (I) reaction time and temperature, (II) ionic liquid loading and temperature and (III) ionic liquid loading and reaction time on (**A**) glucose and (**B**) xylose yield from hemp hurd.

Process parameter effects on the glucose yield and xylose yield of hemp hurd examined the combined effects of reaction time with ionic liquid loading and the combined effect of ionic liquid with reaction time on glucose (Figure 5(A-I–A-III)) and xylose yield (Figure 5(B-I–B-III)). We can observe that the glucose and xylose yields increased with the increased temperature. However, the glucose and xylose yield increased with higher reaction time and ionic liquid loading up to 2 h and 7.5 wt%. After those points, the glucose and xylose yield decreased with the increased reaction time and ionic liquid loading, as observed in the hemp hurd test. ANOVA results shown in Tables S3 and S4 confirmed that reaction temperature had a significant effect on the glucose yield (*p* < 0.0049) and xylose yield (*p* < 0.0024). The interaction effect of reaction temperature with ionic liquid loading and reaction time with ionic liquid loading had a significant effect on glucose yield (*p* < 0.0318 and *p* < 0.0199), while the interaction effect of reaction temperature with reaction time had no statistically significant effect on glucose yield (*p* > 0.0520). Additionally, xylose yields were significantly affected by the combined effect of reaction temperature with reaction time and reaction time with ionic liquid loading (*p* < 0.0278 and *p* < 0.0261). Quadratic effects of ionic liquids on glucose yield and xylose yield were confirmed (*p* < 0.0091 and 0.0065).

In summary, these results demonstrate that the process parameters have different effects on the fermentable sugar yield of hemp hurd compared to the packaging material and implies that mycelium growth affects the hemp hurd material properties. However, no significant improvement in the sugar yield was observed from packaging materials compared to hemp hurd. Even though packaging materials had higher sugar yield in less severe reaction conditions, the highest sugar yield was confirmed from hemp hurd in the harsher reaction condition. Therefore, economic evaluation combined with the evaluation of the reaction condition severity needs to be performed to determine which process parameter condition will be more beneficial. One important consideration is that the packaging materials were less dense than hemp hurd, possibly affecting the degree of mixing of the materials with ionic liquids during pretreatment. Therefore, further investigation of other process parameters such as biomass loading and agitation method is required [34,41,42].

## 3. Materials and Methods

### 3.1. Materials and Chemicals

Hemp hurd and packaging materials were donated by Ecovative Design LLC (Green Island, NY, USA) for evaluation. Both materials were sundried for 24 h and knife-milled with a 2 mm screen (Thomas-Wiley model 4, Swedesboro, NJ, USA). For biomass pretreatment, cholinium lysinate [Ch][Lys] was obtained from Proionic (Grambach, Styria). Commercial cellulase (Cellic CTec3) and hemicellulase (Cellic HTec3) were provided by Novozymes (Franklinton, NC, USA). Sulfuric acid (72% and ACS reagent, >99%), glucose (>99.5%), xylose (>99%), and arabinose (>98%) were purchased from Sigma-Aldrich (St. Louis, MO, USA).

### 3.2. Compositional Analysis

The biomass composition analysis of the hemp hurd and packaging materials was performed to determine glucan, xylan, lignin, and ash contents following the procedure described by NREL [43]. In summary, 0.3 g of biomass was soaked with 3 mL of 72% *w/w* H_2_SO_4_ at 30 °C for 1 h, followed by secondary hydrolysis at 121 °C for 1 h after adding 84 mL of DI water. After the two-step acidic hydrolysis, the mixture was filtered to separate glucan, xylan, and acid-soluble lignin from acid-insoluble lignin through filter crucibles. Acid-insoluble lignin was determined by subtracting the weight of residual solids dried in the oven at 105 °C and the weight of ash formed after burning at 575 °C. Acid-soluble lignin was determined by UV-VIS at 240 nm using a Nanodrop spectrophotometer (Thermo Scientific, Waltham, MA, USA). Monomeric sugars (glucose and xylose) were determined by HPLC using an Agilent 1200 series instrument (Agilent Technologies, Santa Clara, CA, USA) equipped with a refractive index detector and an HPX-87H column (Bio-Rad, Hercules, CA, USA). The instrument was operated at 0.6 mL/min flow rate using 4 mM H_2_SO_4_ as mobile phase and 60 °C column oven temperature. Extractives in the hemp hurd and packaging materials were removed through a water and ethanol extraction process using Dionex ASE 350 (Thermo Scientific, Waltham, MA, USA) following a procedure described by NREL [44]. The extractive content was determined by the dry weight differences before and after the extraction.

### 3.3. Biomass Pretreatment and Enzymatic Hydrolysis

For the comparison between hemp hurd and packaging materials, biomass pretreatment was carried out at a 20 wt% biomass loading in a one-pot (separation-free) configuration. An amount of 1 g of the biomass was mixed thoroughly with 5 g of solvent (10 wt% [Ch][Lys], and 90 wt% water) in a pressure tube (15 mL, Ace Glass Inc., Vineland, NJ, USA). The mixture was heated in an oil bath at 120 °C for 3 h. After pretreatment, the pH of biomass slurry was adjusted to 5 by adding 10 M HCl and 2 M NaOH in preparation for enzymatic hydrolysis. Subsequently, 20 mg of a commercial enzyme mixture (Cellic CTec3 and HTec3, 9:1 *v/v*) per g of biomass was added to the biomass slurry. Enzymatic hydrolysis was conducted at 50 °C for 72 h at 50 rpm in a rotary incubator. After hydrolysis, samples were collected and centrifuged at 4500 rpm for 10 min followed by the filtration of the supernatant with 0.45 µm centrifuge filters before sugar analysis by HPLC. All the pretreatment experiments were performed in triplicate and the standard deviation was estimated to represent errors.

### 3.4. Process Optimization

A Box–Behnken design (JMP Pro 14, SAS Institute, Inc., Cary, NC, USA) was used to explore the effect of each pretreatment process parameter on the saccharification yield and to optimize the pretreatment conditions. A preliminary test was conducted at a temperature of 120 °C, 10% ionic liquid loading and 3 h reaction time, which is the harshest pretreatment condition in our setup. To determine the severity of each process parameter on the yield and identify milder reaction conditions for potentially improving the economic viability of the process, three levels for the factors such as temperature (X_1_), ionic liquid loading (X_2_) and pretreatment time (X_3_) were selected to 100–140 °C, 5–10%, and 1–3 h, respectively (Table 2). Low and high levels of each factor were coded as −1 and +1. A total of 14 experiments were designed and performed in duplicate. Three center points were used to determine the experimental error and reproducibility of the responses. Glucose yield and xylose yield were selected as the response. A secondary-degree polynomial Equation (5) was fitted to the experimental results as a function of the factors for optimizing pretreatment conditions and investigating the effect of parameters, as follows:

Y = β_0_ + β_1_X_1_ + β_2_X_2_ + β_3_X_3_ + β_12_X_1_X_2_ + β_13_X_1_X_3_ + β_23_X_2_X_3_ + β_11_X_1_^2^ + β_22_X_2_^2^ + β_33_X_3_^2^ (5)

where Y is the response, β_0_ is the intercept, β_1_, β_2_, β_3_ and β_12_, β_13_, β_23_ and β_11_, β_22_, β_33_ are linear, interaction and quadratic effect regression coefficients, respectively. The JMP statistical package was used to formulate the design and analyze the obtained data.

### 3.5. Yeast Strain and Cultivation Conditions

An engineered *Rhodosporidium toruloides* strain called GB2 that produces the sesquiterpene bisabolene was used in the bioconversion experiments. Details on strain construction and characterization have been previously reported [33], and the strain is deposited in the Agile BioFoundry public registry https://public-registry.agilebiofoundry.org (accessed on 29 January 2023) under the ID number ABFPUB_000319.

For microbial growth experiments, the pretreated and saccharified hydrolysates were pH-adjusted to 7.5 using 10 N NaOH, supplemented with ammonium sulfate to reach a final concentration of 5 g/L, and filtered through 0.45 µm surfactant-free cellulose-acetate membranes. A fraction of the hydrolysates was diluted 50% by adding water, before pH adjusting, nitrogen supplementation and filtering. For fermentations, cultures were started by adding 1 mL of a frozen glycerol stock to 49 mL of yeast peptone dextrose broth (YPD) in a 500 mL baffled flask and grown at 30 °C and 200 rpm for 24 h. 20 µL of cells in the grown cultures were combined with 780 µL of hydrolysate per reaction in 48-well FlowerPlates (m2p labs, Aachen, Germany). A dodecane overlay (200 µL per well) was added to capture bisabolene from the aqueous phase throughout the fermentation. The plates were covered with sterile AeraSeal films (Excel Scientific, Victorville, CA, USA) and incubated for 7 days in a humidity-controlled incubator with orbital shaking at 999 rpm. At the end of the fermentation, the entire contents of each well were collected in 1.5 mL tubes and centrifuged to separate the overlay, supernatant, and cell fractions. The cell pellets were resuspended in 800 µL of water, diluted with water, and 100 µL per sample were transferred to a Costar black 96-well plate with a flat, clear bottom (Corning, Glendale, AZ, USA) to measure optical density at 600 nm with a SpectraMax Plus 384 reader (Molecular Devices, San Jose, CA, USA). Substrates in the supernatant were analyzed by HPLC with the same method described in Section 3.3. Bisabolene was quantified by GC-MS using previously published methods [39]. The bisabolene concentrations reported here represent the concentrations that would be present in the aqueous phase of the cultivations.

## 4. Conclusions

This work demonstrates the feasibility of hemp hurd and packaging materials made of mycelium grown on hemp hurd to be used as feedstocks for bioconversion to a jet-fuel precursor using a one-pot ionic liquid technology. During the initial test (120 °C, 7.5 wt% ionic liquid loading and 2 h reaction time), the packaging materials produced higher sugar concentrations (43 g/L of glucose and 19.3 g/L of xylose) and yield (80.4% for glucose and 87.1% for xylose) than the hemp hurd (35.2 g/L of glucose and 16.2 g/L of xylose and 66.4% and 68.3% glucose and xylose yield, respectively). However, the Box–Behnken experimental design showed that the reaction conditions for the maximum sugar yields from each material was different and that the significance of the process parameter effect on the fermentable sugar yield was dependent on the biomass properties, suggesting that the mycelial growth affected the deconstructability of the hemp hurd. Furthermore, the fermentation test to convert fermentable sugar into bisabolene showed that hydrolysates from the packaging material resulted in a higher bisabolene titer (1400 mg/L) than hydrolysates from the hemp hurd, probably due to the higher sugar concentrations generated form the packaging material.

To fully take advantage of these packaging materials to produce biofuels after they are used and discarded, a more detailed correlation study between the fermentable sugar yield and physicochemical properties of biomass and packaging materials or packaging process parameters is required by testing different hemp material sources. In addition, methods to overcome hydrolysate toxicity will need to be employed to enable utilization of concentrated hydrolysate for increased product titers and a reduction in water consumption. Finally, further investigation into other process parameters such as agitation and biomass loadings are merited to fully optimize the pretreatment conditions, as well as performing pilot scale tests to generate data that can help assess the economic feasibility of this new conceptual process. Overall, this study indicates that it is possible to produce lignocellulosic supply chains for production of biofuels and biochemicals that include both raw biomass and biomass that has been first processed and valorized as commercial products, such as packaging materials, enabling the carbon in these lignocellulosic products to generate value multiple times in their life cycle.

## Figures and Tables

**Table 1:** Chemical composition of hemp hurd and packaging materials.

	Hemp Hurd (wt%)	Packaging Material (wt%)
Extractives	8.3 ± 3.2	14.7 ± 1.1
Glucan	30.3 ± 0.9	28.6 ± 0.1
Xylan	13.5 ± 0.5	11.9 ± 0.1
Klason lignin	22.4 ± 0.7	22.4 ± 3.0
Ash	0.6 ± 0.4	0.6 ± 0.3

**Table 2:** Experimental variables and their code levels in the Box–Behnken design.

Variables	Factor Code	Level of Factor		
		−1	0	1
Temperature (°C)	X1	100	120	140
Time (h)	X2	1	2	3
Ionic liquid loading (%)	X3	5	7.5	10

## Data Availability

All data are contained within the article and Supplementary Materials.

## Supplementary Materials

The following supporting information can be downloaded at: https://www.mdpi.com/article/10.3390/molecules28031427/s1. Table S1: Glucose yields from hydrolysis of one-pot pretreated hemp hurd and packaging material under different experimental conditions. Table S2: Xylose yields from hydrolysis of one-pot pretreated hemp hurd and packaging material under different experimental conditions. Table S3: ANOVA, summary of fit and significance of regression coefficients for glucose yield model of hemp hurd. Table S4: ANOVA, summary of fit and significance of regression coefficients for xylose yield model of hemp hurd. Table S5: ANOVA, summary of fit and significance of regression coefficients for glucose yield model of packaging material. Table S6: ANOVA, summary of fit and significance of regression coefficients for xylose yield model of packaging material.
