# Supplementary material for: Valorization of Hemp-Based Packaging Waste with One-Pot Ionic Liquid Technology
Source: Molecules. 2023 Feb 2;28(3):1427. doi: 10.3390/molecules28031427 (PMC9919018; doi:10.3390/molecules28031427)
Supplement: Supplementary file 1 [file molecules-28-01427-s001.zip › Table S1.docx]

**Table S1.** Glucose yields from hydrolysis of one-pot pretreated hemp hurd and packaging material under different experimental conditions.

| Run | Coded factor | | |  | Hemp hurd | | |  | Packaging material | | |
| --- | --- | --- | --- | --- | --- | --- | --- | --- | --- | --- | --- |
|  | X1 | X2 | X3 |  | Actual data | Predicted data | Residual |  | Actual data | Predicted data | Residual |
| 1 | -1 | -1 | 0 |  | 28.2 | 33.51 | -5.31 |  | 51.2 | 50.25 | 0.95 |
| 2 | 1 | -1 | 0 |  | 81.6 | 85.91 | -4.31 |  | 69.4 | 68.05 | 1.35 |
| 3 | -1 | 1 | 0 |  | 72.5 | 68.19 | 4.31 |  | 60.8 | 62.15 | -1.35 |
| 4 | 1 | 1 | 0 |  | 77.1 | 71.79 | 5.31 |  | 64.3 | 65.25 | -0.95 |
| 5 | 0 | 0 | 0 |  | 68 | 68.43 | -0.43 |  | 75 | 73.53 | 1.47 |
| 6 | 0 | -1 | -1 |  | 46.4 | 37.28 | 9.13 |  | 59.9 | 61.95 | -2.05 |
| 7 | 0 | 1 | -1 |  | 49.3 | 49.8 | -0.5 |  | 68.9 | 68.65 | 0.25 |
| 8 | 0 | -1 | 1 |  | 25.3 | 24.8 | 0.5 |  | 61.8 | 62.05 | -0.25 |
| 9 | 0 | 1 | 1 |  | 23.7 | 32.83 | -9.13 |  | 66.5 | 64.45 | 2.05 |
| 10 | 0 | 0 | 0 |  | 67 | 68.43 | -1.43 |  | 73 | 73.53 | -0.53 |
| 11 | -1 | 0 | -1 |  | 56.3 | 60.11 | -3.81 |  | 61 | 59.90 | 1.10 |
| 12 | 1 | 0 | -1 |  | 73 | 77.81 | -4.81 |  | 67.5 | 66.80 | 0.70 |
| 13 | -1 | 0 | 1 |  | 39.9 | 35.09 | 4.81 |  | 53.6 | 54.30 | -0.70 |
| 14 | 1 | 0 | 1 |  | 77.2 | 73.39 | 3.81 |  | 67.2 | 68.30 | -1.10 |
| 15 | 0 | 0 | 0 |  | 70.3 | 68.43 | 1.87 |  | 72.6 | 73.53 | -0.93 |
