# Supplementary material for: Valorization of Hemp-Based Packaging Waste with One-Pot Ionic Liquid Technology
Source: Molecules. 2023 Feb 2;28(3):1427. doi: 10.3390/molecules28031427 (PMC9919018; doi:10.3390/molecules28031427)
Supplement: Supplementary file 1 [file molecules-28-01427-s001.zip › Table S2.docx]

**Table S2.** Xylose yields from hydrolysis of one-pot pretreated hemp hurd and packaging material under different experimental conditions.

| Run | Coded factor | | |  | Hemp hurd | | |  | Packaging material | | |
| --- | --- | --- | --- | --- | --- | --- | --- | --- | --- | --- | --- |
|  | X1 | X2 | X3 |  | Actual data | Predicted data | Residual |  | Actual data | Predicted data | Residual |
| 1 | -1 | -1 | 0 |  | 34.2 | 37.86 | -3.66 |  | 51.8 | 55.19 | -3.39 |
| 2 | 1 | -1 | 0 |  | 90.5 | 91.46 | -0.96 |  | 76.2 | 72.14 | 4.06 |
| 3 | -1 | 1 | 0 |  | 65.8 | 64.84 | 0.96 |  | 63.5 | 67.56 | -4.06 |
| 4 | 1 | 1 | 0 |  | 82.5 | 78.84 | 3.66 |  | 74 | 70.61 | 3.39 |
| 5 | 0 | 0 | 0 |  | 74.3 | 68.50 | 5.80 |  | 81 | 80.40 | 0.60 |
| 6 | 0 | -1 | -1 |  | 42.3 | 34.43 | 7.88 |  | 57.1 | 57.98 | -0.87 |
| 7 | 0 | 1 | -1 |  | 49.8 | 46.55 | 3.25 |  | 69.2 | 69.40 | -0.20 |
| 8 | 0 | -1 | 1 |  | 25.1 | 28.35 | -3.25 |  | 69.5 | 69.30 | 0.20 |
| 9 | 0 | 1 | 1 |  | 22.7 | 30.58 | -7.88 |  | 69.6 | 68.73 | 0.88 |
| 10 | 0 | 0 | 0 |  | 66.7 | 68.50 | -1.80 |  | 80 | 80.40 | -0.40 |
| 11 | -1 | 0 | -1 |  | 50.9 | 55.11 | -4.21 |  | 64 | 59.74 | 4.26 |
| 12 | 1 | 0 | -1 |  | 73.5 | 80.41 | -6.91 |  | 70.7 | 73.89 | -3.19 |
| 13 | -1 | 0 | 1 |  | 42.5 | 35.59 | 6.91 |  | 72.4 | 69.21 | 3.19 |
| 14 | 1 | 0 | 1 |  | 82.1 | 77.89 | 4.21 |  | 70.8 | 75.06 | -4.26 |
| 15 | 0 | 0 | 0 |  | 64.5 | 68.50 | -4.00 |  | 80.2 | 80.40 | -0.20 |
