# Supplementary material for: Valorization of Hemp-Based Packaging Waste with One-Pot Ionic Liquid Technology
Source: Molecules. 2023 Feb 2;28(3):1427. doi: 10.3390/molecules28031427 (PMC9919018; doi:10.3390/molecules28031427)
Supplement: Supplementary file 1 [file molecules-28-01427-s001.zip › Table S3.docx]

**Table S3.** ANOVA, summary of fit and significance of regression coefficients for glucose yield model of hemp hurd.

| ANOVA | | | | | | | | | |
| --- | --- | --- | --- | --- | --- | --- | --- | --- | --- |
|  | Degree of freedom | | Sum of Squares | | Mean Square | | F Ratio | | Prob > F |
| Model | 9 | | 0.538 | | 0.0597 | | 8.7404 | | 0.014 |
| Error | 5 | | 0.0342 | | 0.00684 | |  | |  |
| C.Total | 14 | | 0.572 | |  | |  | |  |
| Summary of Fit | | | | | | | | | |
| RSquare | | | | | 0.940237 | | | | |
| RSquare Adj | | | | | 0.832664 | | | | |
| Root Mean Square Error | | | | | 0.082679 | | | | |
| Mean of Response | | | | | 0.570533 | | | | |
| Observations (or Sum Wgts) | | | | | 15 | | | | |
| Significance of regression coefficients | | | | | | | | | |
| Term | | Estimates | | Std. Error | | t Ratio | | Prob > t | |
| Intercept | | -0.037542 | | 0.210114 | | -0.18 | | 0.8652 | |
| X_1_ | | 0.007 | | 0.001462 | | 4.79 | | 0.0049* | |
| X_2_ | | 0.051375 | | 0.029231 | | 1.76 | | 0.1392 | |
| X_3_ | | -0.02945 | | 0.011693 | | -2.52 | | 0.0533 | |
| X_1_X_2_ | | 0.000273 | | 0.000108 | | 2.54 | | 0.0520 | |
| X_1_X_3_ | | -0.0061 | | 0.002067 | | -2.95 | | 0.0318* | |
| X_2_X_3_ | | -0.145042 | | 0.043027 | | -3.37 | | 0.0199* | |
| X_1_^2^ | | 0.00103 | | 0.000827 | | 1.25 | | 0.2680 | |
| X_2_^2^ | | -0.0045 | | 0.016536 | | -0.27 | | 0.7964 | |
| X_3_^2^ | | -0.028407 | | 0.006884 | | -4.13 | | 0.0091* | |
