# Supplementary material for: Valorization of Hemp-Based Packaging Waste with One-Pot Ionic Liquid Technology
Source: Molecules. 2023 Feb 2;28(3):1427. doi: 10.3390/molecules28031427 (PMC9919018; doi:10.3390/molecules28031427)
Supplement: Supplementary file 1 [file molecules-28-01427-s001.zip › Table S4.docx]

**Table S4.** ANOVA, summary of fit and significance of regression coefficients for xylose yield model of hemp hurd.

| ANOVA | | | | | | | | | |
| --- | --- | --- | --- | --- | --- | --- | --- | --- | --- |
|  | Degree of freedom | | Sum of Squares | | Mean Square | | F Ratio | | Prob > F |
| Model | 9 | | 0.606 | | 0.0674 | | 9.4149 | | 0.0118 |
| Error | 5 | | 0.0358 | | 0.00716 | |  | |  |
| C.Total | 14 | | 0.642 | |  | |  | |  |
| Summary of Fit | | | | | | | | | |
| RSquare | | | | | 0.94428 | | | | |
| RSquare Adj | | | | | 0.843984 | | | | |
| Root Mean Square Error | | | | | 0.08459 | | | | |
| Mean of Response | | | | | 0.578267 | | | | |
| Observations (or Sum Wgts) | | | | | 15 | | | | |
| Significance of regression coefficients | | | | | | | | | |
| Term | | Estimates | | Std. Error | | t Ratio | | Prob > t | |
| Intercept | | -0.235375 | | 0.21497 | | -1.09 | | 0.3235 | |
| X_1_ | | 0.00845 | | 0.001495 | | 5.65 | | 0.0024* | |
| X_2_ | | 0.035875 | | 0.029907 | | 1.20 | | 0.2841 | |
| X_3_ | | -0.02205 | | 0.011963 | | -1.84 | | 0.1246 | |
| X_1_X_2_ | | 0.0003378 | | 0.00011 | | 3.07 | | 0.0278* | |
| X_1_X_3_ | | -0.00495 | | 0.002115 | | -2.34 | | 0.0663 | |
| X_2_X_3_ | | -0.137625 | | 0.044022 | | -3.13 | | 0.0261* | |
| X_1_^2^ | | 0.00085 | | 0.000846 | | 1.00 | | 0.3611 | |
| X_2_^2^ | | -0.0099 | | 0.016918 | | -0.59 | | 0.5838 | |
| X_3_^2^ | | -0.03162 | | 0.007044 | | -4.49 | | 0.0065* | |
