# Supplementary material for: Valorization of Hemp-Based Packaging Waste with One-Pot Ionic Liquid Technology
Source: Molecules. 2023 Feb 2;28(3):1427. doi: 10.3390/molecules28031427 (PMC9919018; doi:10.3390/molecules28031427)
Supplement: Supplementary file 1 [file molecules-28-01427-s001.zip › Table S5.docx]

**Table S5.** ANOVA, summary of fit and significance of regression coefficients for glucose yield model of packaging material.

| ANOVA | | | | | | | | | |
| --- | --- | --- | --- | --- | --- | --- | --- | --- | --- |
|  | Degree of freedom | | Sum of Squares | | Mean Square | | F Ratio | | Prob > F |
| Model | 9 | | 0.0639 | | 0.00711 | | 17.1718 | | 0.003 |
| Error | 5 | | 0.00207 | | 0.000414 | |  | |  |
| C.Total | 14 | | 0.0660 | |  | |  | |  |
| Summary of Fit | | | | | | | | | |
| RSquare | | | | | 0.968661 | | | | |
| RSquare Adj | | | | | 0.912251 | | | | |
| Root Mean Square Error | | | | | 0.02034 | | | | |
| Mean of Response | | | | | 0.648467 | | | | |
| Observations (or Sum Wgts) | | | | | 15 | | | | |
| Significance of regression coefficients | | | | | | | | | |
| Term | | Estimates | | Std. Error | | t Ratio | | Prob > t | |
| Intercept | | 0.407 | | 0.0517 | | 7.88 | | 0.0005* | |
| X_1_ | | 0.00261 | | 0.00036 | | 7.27 | | 0.0008* | |
| X_2_ | | 0.0228 | | 0.00719 | | 3.16 | | 0.0250* | |
| X_3_ | | -0.0041 | | 0.00288 | | -1.43 | | 0.2134 | |
| X_1_X_2_ | | -0.000176 | | 2.65e-5 | | -6.64 | | 0.0012* | |
| X_1_X_3_ | | -0.00184 | | 0.000509 | | -3.61 | | 0.0153* | |
| X_2_X_3_ | | -0.0508 | | 0.0106 | | -4.80 | | 0.0049* | |
| X_1_^2^ | | 0.000355 | | 0.000203 | | 1.75 | | 0.1414 | |
| X_2_^2^ | | -0.0043 | | 0.00407 | | -1.06 | | 0.3389 | |
| X_3_^2^ | | -0.00669 | | 0.00169 | | -3.95 | | 0.0109* | |
