# Supplementary material for: Valorization of Hemp-Based Packaging Waste with One-Pot Ionic Liquid Technology
Source: Molecules. 2023 Feb 2;28(3):1427. doi: 10.3390/molecules28031427 (PMC9919018; doi:10.3390/molecules28031427)
Supplement: Supplementary file 1 [file molecules-28-01427-s001.zip › Table S6.docx]

**Table S6.** ANOVA, summary of fit and significance of regression coefficients for xylose yield model of packaging material.

| ANOVA | | | | | | | | | |
| --- | --- | --- | --- | --- | --- | --- | --- | --- | --- |
|  | Degree of freedom | | Sum of Squares | | Mean Square | | F Ratio | | Prob > F |
| Model | 9 | | 0.0849 | | 0.00943 | | 4.1068 | | 0.0671 |
| Error | 5 | | 0.0115 | | 0.00230 | |  | |  |
| C.Total | 14 | | 0.0963 | |  | |  | |  |
| Summary of Fit | | | | | | | | | |
| RSquare | | | | | 0.880842 | | | | |
| RSquare Adj | | | | | 0.666357 | | | | |
| Root Mean Square Error | | | | | 0.047914 | | | | |
| Mean of Response | | | | | 0.7 | | | | |
| Observations (or Sum Wgts) | | | | | 15 | | | | |
| Significance of regression coefficients | | | | | | | | | |
| Term | | Estimates | | Std. Error | | t Ratio | | Prob > t | |
| Intercept | | 0.370 | | 0.122 | | 3.04 | | 0.0288* | |
| X_1_ | | 0.0025 | | 0.000847 | | 2.95 | | 0.0318* | |
| X_2_ | | 0.0271 | | 0.0169 | | 1.60 | | 0.1702 | |
| X_3_ | | 0.0107 | | 0.00678 | | 1.57 | | 0.1768 | |
| X_1_X_2_ | | -0.000136 | | 6.23e-5 | | -2.19 | | 0.0805 | |
| X_1_X_3_ | | -0.00174 | | 0.00120 | | -1.45 | | 0.2066 | |
| X_2_X_3_ | | -0.0858 | | 0.0249 | | -3.44 | | 0.0185* | |
| X_1_^2^ | | -0.000415 | | 0.000479 | | -0.87 | | 0.4260 | |
| X_2_^2^ | | -0.012 | | 0.00958 | | -1.25 | | 0.2659 | |
| X_3_^2^ | | -0.00876 | | 0.00399 | | -2.20 | | 0.0795 | |
